# Correction: The mediating role of workplace milieu resources on the relationship between emotional intelligence and burnout among leaders in social care

**DOI:** 10.1371/journal.pone.0334105

**Published:** 2025-10-08

**Authors:** Anna Kozák, Réka Schutzmann, Klára Soltész-Várhelyi, Fruzsina Albert

The affiliation for the fourth author is incorrect. The correct affiliations are not indicated. Fruzsina Albert is not affiliated with #1 but with: Institute for Sociology, HUN-REN Centre for Social Sciences, Hungary and Institute of Mental Health, Semmelweis University, Hungary.
