# Supplementary material for: The mediating role of workplace milieu resources on the relationship between emotional intelligence and burnout among leaders in social care
Source: PLoS One. 2025 Jan 31;20(1):e0317280. doi: 10.1371/journal.pone.0317280 (PMC11785285; doi:10.1371/journal.pone.0317280)
Supplement: S1 File — (DOCX) [file pone.0317280.s001.docx]

**S1 Survey Questionnaire Items**

| **Demographic Questions** |
| --- |
| Gender |
| Year of Birth |
| Level of Education |
| Years of full-time job experience |
| Municipality type of the workplace |
| Number of leadership years |
| Number of subordinates |
| **The Schutte Emotional Intelligence Test The 33-item emotional intelligence scale** |
| 1. I know when to speak about my personal problems to others |
| 2. When I am faced with obstacles, I remember times I faced similar obstacles and overcame them |
| 3. I expect that I will do well on most things I try |
| 4. Other people find it easy to confide in me |
| 5. I find it hard to understand the non-verbal messages of other people* |
| 6. Some of the major events of my life have led me to re-evaluate what is important and not important |
| 7. When my mood changes, I see new possibilities |
| 8. Emotions are one of the things that make my life worth living |
| 9. I am aware of my emotions as I experience them |
| 10. I expect good things to happen |
| 11. I like to share my emotions with others |
| 12. When I experience a positive emotion, I know how to make it last |
| 13. I arrange events others enjoy |
| 14. I seek out activities that make me happy |
| 15. I am aware of the non-verbal messages I send to others |
| 16. I present myself in a way that makes a good impression on others |
| 17. When I am in a positive mood, solving problems is easy for me |
| 18. By looking at their facial expressions, I recognize the emotions people are experiencing |
| 19. I know why my emotions change |
| 20. When I am in a positive mood, I am able to come up with new ideas |
| 21. I have control over my emotions |
| 22. I easily recognize my emotions as I experience them |
| 23. I motivate myself by imagining a good outcome to tasks I take on |
| 24. I compliment others when they have done something well |
| 25. I am aware of the non-verbal messages other people send |
| 26. When another person tells me about an important event in his or her life, I almost feel as though I have |
| experienced this event myself |
| 27. When I feel a change in emotions, I tend to come up with new ideas |
| 28. When I am faced with a challenge, I give up because I believe I will fail* |
| 29. I know what other people are feeling just by looking at them |
| 30. I help other people feel better when they are down |
| 31. I use good moods to help myself keep trying in the face of obstacles |
| 32. I can tell how people are feeling by listening to the tone of their voice |
| 33. It is difficult for me to understand why people feel the way they do* |
| **Questions from the COPSOQ II. Middle version** |
| **Burnout scale** |
| How often have you felt worn out? |
| How often have you been physically exhausted? |
| How often have you been emotionally exhausted? |
| How often have you felt tired? |
| **The sense of community (labelled as social community at work in COPSOQ II) scale** |
| Is there a good atmosphere between you and your colleagues? |
| Is there good cooperation between the colleagues at work? |
| Do you feel part of a community at your place of work? |
| **Mutual Trust Between Employees scale** |
| Do the employees withhold information from each other? |
| Do the employees withhold information from the management? |
| Do the employees, in general, trust each other? |
