# Supplementary material for: The mediating role of workplace milieu resources on the relationship between emotional intelligence and burnout among leaders in social care
Source: PLoS One. 2025 Jan 31;20(1):e0317280. doi: 10.1371/journal.pone.0317280 (PMC11785285; doi:10.1371/journal.pone.0317280)
Supplement: S2 File — (DOCX) [file pone.0317280.s002.docx]

**S2 Results and loadings of Principal Component Analysis of EI items after Direct Oblimin rotation.**

| **EI-Items** | **EI-positivity** | **EI-others** | **EI-self** | **PC4** | **PC5** |
| --- | --- | --- | --- | --- | --- |
| **10** I expect good things to happen | -.753 |  |  |  |  |
| **12** When I experience a positive emotion, I know how to make it last | -.703 |  |  |  |  |
| **3** I expect that I will do well on most things I try | -.527 |  |  |  |  |
| **31** I use good moods to help myself keep trying in the face of obstacles | -.518 |  |  |  |  |
| **14** I seek out activities that make me happy | -.456 |  |  | .431 |  |
| **24** I compliment others when they have done something well | -.441 |  |  |  |  |
| **1** I know when to speak about my personal problems to others | -.439 |  |  |  |  |
| **4** Other people find it easy to confide in me | -.436 |  |  |  |  |
| **2** When I am faced with obstacles, I remember times I faced similar obstacles and overcame them | -.433 |  |  |  |  |
| **29** I know what other people are feeling just by looking at them |  | .691 |  |  |  |
| **32** I can tell how people are feeling by listening to the tone of their voice |  | .678 |  |  |  |
| **30** I help other people feel better when they are down |  | .588 |  |  |  |
| **26** When another person tells me about an important event in his or her life, I almost feel as though I have experienced this event myself |  | .552 |  |  |  |
| **25** I am aware of the non-verbal messages other people send |  | .543 |  |  |  |
| **18** By looking at their facial expressions, I recognize the emotions people are experiencing |  | .526 |  |  |  |
| **19** I know why my emotions change |  |  | -.599 |  |  |
| **22** I easily recognize my emotions as I experience them |  |  | -.583 |  |  |
| **15** I am aware of the non-verbal messages I send to others |  |  | -.582 |  |  |
| **20** When I am in a positive mood, I am able to come up with new ideas |  |  | -.490 |  |  |
| **17** When I am in a positive mood, solving problems is easy for me |  |  | -.473 | .431 |  |
| **9** I am aware of my emotions as I experience them |  |  | -.441 |  |  |
| **8** Emotions are one of the things that make my life worth living |  |  |  | .682 |  |
| **7** When my mood changes, I see new possibilities |  |  |  | .666 |  |
| **6** Some of the major events of my life have led me to re-evaluate what is important and not important |  |  |  | .599 |  |
| **33** It is difficult for me to understand why people feel the way they do |  |  |  |  | -.545 |
| **16** I present myself in a way that makes a good impression on others |  |  |  |  | .493 |
| **28** When I am faced with a challenge, I give up because I believe I will fail |  |  |  |  | -.466 |
| **23** I motivate myself by imagining a good outcome to tasks I take on |  |  |  |  | .451 |
| **5** I find it hard to understand the non-verbal messages of other people |  |  |  |  | -.433 |
| **Eigenvalue after rotation** | 5.12 | 5.05 | 4.235 | 2.844 | 2.333 |
| **Percentage of variance after rotation** | 15.515 | 15.303 | 12.833 | 8.618 | 7.069 |

Note. Data reduction method: Principal Component Analysis. Rotation method: Direct Oblimin with Kaiser Normalization; Component’s loadings > .04 were displayed in the table. N = 528. KMO = .889; and for Bartlett’s test *χ²*(528) = 4889.529 *p* < .001. Total variance explained: 45.8%. Scales were re-standardized using the mean and standard deviation of the subsample used in the mediation analyses.
