# Supplementary material for: The mediating role of workplace milieu resources on the relationship between emotional intelligence and burnout among leaders in social care
Source: PLoS One. 2025 Jan 31;20(1):e0317280. doi: 10.1371/journal.pone.0317280 (PMC11785285; doi:10.1371/journal.pone.0317280)
Supplement: S3 File — (DOCX) [file pone.0317280.s003.docx]

**S3 Skewness and Kurtosis values of relevant variables**

| **Variables** | **Skewness** | **SE of Skewness** | **Kurtosis** | **SE of Kurtosis** |
| --- | --- | --- | --- | --- |
| EIS | -0.29 | 0.11 | 0.76 | 0.22 |
| EIO | -0.12 | 0.11 | 0.18 | 0.22 |
| EIP | -0.63 | 0.11 | 1.12 | 0.22 |
| Sense of Community | -0.92 | 0.11 | 2.02 | 0.22 |
| Mutual Trust between employees | -0.06 | 0.11 | -0.28 | 0.22 |
| Burnout | 0.13 | 0.11 | -0.18 | 0.22 |
