# Supplementary material for: The mediating role of workplace milieu resources on the relationship between emotional intelligence and burnout among leaders in social care
Source: PLoS One. 2025 Jan 31;20(1):e0317280. doi: 10.1371/journal.pone.0317280 (PMC11785285; doi:10.1371/journal.pone.0317280)
Supplement: S4 File — (DOCX) [file pone.0317280.s004.docx]

**S4 Lavann Syntax**

#EIX should be changed to EIS, EIO and EIP in case of EI-self, EI-others and EI-positivity, accordinly.

# dependent regression

burnout ~ b1*community + b2*trust + cprime*EIX

# mediator regression

community ~ a1*EIX

trust ~ a2*EIX + d*community

# confounder adjustment

EIX ~ age + gender

community ~ age + gender

trust ~ age + gender

burnout ~ age + gender

# indirect effects

ind_x_m1_y := a1*b1

ind_x_m2_y := a2*b2

ind_x_m1_m2_y := a1*d*b2

indtotal := ind_x_m1_y + ind_x_m2_y + ind_x_m1_m2_y

ctotal := indtotal + cprime
