# Supplementary material for: The mediating role of workplace milieu resources on the relationship between emotional intelligence and burnout among leaders in social care
Source: PLoS One. 2025 Jan 31;20(1):e0317280. doi: 10.1371/journal.pone.0317280 (PMC11785285; doi:10.1371/journal.pone.0317280)
Supplement: Attachment — Submitted filename: Response to Reviewers_18_10.docx [file pone.0317280.s006.docx]

**Dear Editor and Reviewers,**

We sincerely appreciate your valuable comments and insights on our manuscript. We have thoroughly reviewed all your suggestions and made comprehensive amendments to enhance the document. We believe these revisions have significantly improved its quality. The changes proposed by the reviewers are detailed in the table format required in the PLOS ONE Revision letter.

**Below are our responses to the journal's requirements:**

1. **Manuscript Style Requirements**: We appreciate your feedback. We have thoroughly reviewed the style requirements once more to ensure our manuscript meets all guidelines.

2. **Data Sharing Policy**: We have established the database and will upload it to one of the recommended repositories upon acceptance of the manuscript.

3. **Supplementary Tables**: We have created new supplementary documents, and all tables and supporting information have been relocated there. Additionally, we have ensured that a legend list for all supplementary materials is included in the manuscript.

**Authors Comments to the Reviewers**

**Reviewer1**

| **Reviewers' Comment** | **Authors revision** |
| --- | --- |
| The abstract needs methods and analysis techniques, such as administering only surveys. | Thank you for your statement. We have extended and adjusted the abstract as required. |
| The analysis uses SPSS and SEM, such as AMOS, Smart PLS, and ADANCO. | Thank you for your statement. However, we would like to clarify that our article does not reference the tools or methods you mentioned. As the Data Analysis section outlines, the analyses were conducted using JASP. The data analysis section was adjusted to clarify that we used a saturated model of serial mediation. The parameterization of these analyses was also added to this section. |
| The introduction could be more comprehensive. | Thank you very much for your suggestion. We have extended the introduction and made it more comprehensive. |
| A literature review is necessary for defining terms including all variables in this study based on theories, and research hypotheses. | Thank you very much for your comments. We have entered hypotheses based on the assumptions and facts of the literature review. For emotional intelligence, we extended the definition with the main components of the construct as follows: "Nevertheless the difference between the theories the facets are similar because of the dominance of Salovey and Mayer's theory. The four more widely used facets or constructs are the following: "(1) perceiving emotions (in self and others), (2) regulating emotions in self, (3) regulating emotions in others, and (4) strategically utilizing emotions" [25, pp.3].." For all concepts: EI, Burnout, JD-R mutual trust and sense of community, the definitions have been included in the text so far as follows: **Burnout:** "One of the most widely accepted definitions of burnout is Maslach and Leiter (2016), which defines burnout as "a psychological syndrome that develops as a prolonged response to chronic interpersonal stressors at work" [13, p. 104] (Manuscript p. 5). **EI:** "Salovey and Mayer defined emotional intelligence as "the ability to observe one's own and others' feelings and emotions, to distinguish between them, and to use this information to guide one's thoughts and actions" [24, p. 189] (Manuscript p. 5). **JD-R**: Workplace resources include "physical, psychological, social, or organizational aspects of work that are either: (1) functional in achieving work goals; (2) reduce job demands and associated physiological and psychological costs; (3) promote personal growth and development" [30, 344. On the other hand, demands are "physical, psychological, social or organisational aspects of work that require sustained physical and/or psychological (cognitive and emotional) effort and therefore have certain physiological and/or psychological costs" [30, p. 344]. **Sense of community:** "According to Sarason's original definition, a sense of community is "a sense of similarity to others, a recognized interdependence with others, a willingness to maintain this interdependence by giving to or doing for others what is expected of us, and a sense of being part of a larger, reliable and stable structure"' [35, p. 157] (Manuscript p. 8.) **Trust:** "Although the definition of trust is still debated [39], many researchers accept the definition proposed by Mayer et al. which defines trust as "the willingness of a party to be vulnerable to the actions of another party based on the expectation that the other party will perform a certain action important to the trust-giver, regardless of his or her ability to control or direct the other party" [44, p. 712] (Manuscript p. 8).  **Trust:** "Although the definition of trust is still debated [39], many researchers accept the definition proposed by Mayer et al. which defines trust as "the willingness of a party to be vulnerable to the actions of another party based on the expectation that the other party will perform a certain action important to the trust-giver, regardless of his or her ability to control or direct the other party" [44, p. 712] (Manuscript p. 8). |
| The research objective (aim) is suggested to move to the introduction and add research questions. | Thank you for your valuable feedback. The chapter outlining the research aim has been removed, and the explanatory text has been repositioned to follow the literature review.  Additionally, four hypotheses have been proposed, substantiated by the findings from the literature review as follows:  **H1:** Emotional intelligence negatively affects burnout;  **H2**: Emotional intelligence positively influences a sense of community and indirectly impacts trust between employees;  **H3:** Sense of community and mutual trust between employees negatively affect burnout;  **H4**: Workplace milieu resources mediate the relationship between emotional intelligence and burnout.  Our theoretical model based on these assumptions is presented in Fig 1. |
| Research design is necessary for population, sampling technique, data collection, and data analysis (SPSS for descriptive analysis in frequency, percentage, and mean). | Thank you for the comment. Although the sampling procedure was already included in the original manuscript, it was incorrectly placed in the Participants section. We moved it to the Research Design and Sampling Procedures section and further supplemented it, as you can see at the beginning of the section (pp. 11-12). Descriptive statistics have already been included in the Participant section, but we have supplemented this with additional informative indicators. The descriptive statistics of the relevant indicators are included at the beginning of the text of the Results section and in Table 1. |
| The inferential statistical analysis of PLS-SEM is necessary to explain. | Thank you for the comment. The Data Analysis section was supplemented with a description of the serial mediation used in the study. We emphasized that we used a saturated model and stated that we used percentile bootstrap to estimate the standard errors and confidence intervals used for inferential statistics. |
| For data analysis in Table 2, please add *significant level at p<0.05, **significant level at p<0.01, and ***significant level at p<0.001. | Thank you for the comment, we have added the asterisks and the notes to Table 2 and consequently to Table 3 and Table 4. |
| Discussions are needed to follow the research hypotheses | Thank you for your insightful feedback regarding the discussion section. A concise summary of the results has been incorporated to align with the logical framework of the hypotheses. |
| More tables are required for assumptions. | Thank you for the suggestion, the descriptive statistics used to check the assumptions have been tabulated and placed in the Supplementing information. |
| Avoid using we, but use the researchers instead. | Thank you for your feedback. The sentences that commenced with or included "we" have been rephrased for greater academic clarity. |
| When research hypotheses are unclear, discussions and conclusions are also not clear. The conclusion suggests how a strategic planner could improve the outcome based on predictors. | Research hypotheses have been incorporated, leading to a rephrasing of the discussion section to ensure alignment with these hypotheses. Additionally, future strategic policy implications and directions have been included to enhance the overall analysis. |

**Reviewer2**

| **Reviewers' Comment** | **Author(s) revision** |
| --- | --- |
| When using Grammarly to check AI generated document similarity was found that 49% of this manuscript is similar to AI-generated documents. Thus, the reviewer suggest the authors revise the manuscript to be plagiarism and AI-similarity to be 15% or less. | We thank Reviewer 2 for his/her comment. We would like to inform Reviewer 2 that no original text was created using AI assistance. During the writing, sometimes we used DeepL and Google Translate for the translation (but always only short parts of about one sentence at a time were translated); we checked and corrected the grammar using Grammarly, and our article was proofread by the official English proofreading office of Semmelweis University (we can provide correspondence confirming this if required). However, we, the authors, are not native English speakers. Liang et al. (2023) found that AI detectors discriminate negatively against texts written by non-native English speakers (due to their more straightforward, predictable, rigid sentence structures). Therefore, while native English texts were judged relatively accurately by AI detectors, non-native texts had a high false positive rate. We think this may be the reason for the misunderstanding.  Based on the comments of the other reviewers, the manuscript underwent a significant revision, during which we tried to rely even less on Google Translate and Grammarly, and we hope that the text will become acceptable. However, to further tweak the text to get a low hit value on the AI detector, we find this approach fundamentally dishonest. In addition, we added a paragraph at the end of the manuscript in which we stated the use of Google Translate, Grammarly, DeepL, and professional proofreading. We can only trust in the goodwill of Reviewer 2. Liang et al (2023): https://arxiv.org/pdf/2304.02819 |

**Reviewer3**

| **Reviewers' Comment** | **Authors revision** |
| --- | --- |
| First suggestion, in the abstract there is no explanation of the methodology used, this is very important to be explained. | Thank you for your statement we have extended and adjusted the abstract as required. |
| Second suggestion, to add an in-depth discussion of the problems and empirical gaps that occur so that this research has good useful value | Thank you for your comment. We have added the following paragraph to the end of the introduction:  "This study offers novel insights into how emotional intelligence influences burnout. It focuses on social care leaders in Hungary, a group vulnerable to burnout who also faces unique organizational and bureaucratic burdens in Eastern Europe. By highlighting workplace milieu resources as mediators, the research suggests that emotionally intelligent leaders are better equipped to use social resources at the workplace to mitigate burnout. Thus, the results of this study could further support tailored social policy interventions to improve social care leaders' mental health and working conditions." |
| The third suggestion, make hypothesis development on each influence relationship with relevant articles that support the research framework, because this research uses quantitative methods so it is mandatory to make hypotheses and will be answered in the discussion section. | Thank you for your valuable feedback.   Four hypotheses have been proposed, substantiated by the findings from the literature review as follows:  H1: Emotional intelligence negatively affects burnout;  H2: Emotional intelligence positively influences a sense of community and indirectly impacts trust between employees;  H3: Sense of community and mutual trust between employees negatively affect burnout;  H4: Workplace milieu resources mediate the relationship between emotional intelligence and burnout.  Our theoretical model based on these assumptions is presented in Fig 1. The hypothesis is detailed and supported with literature in the new Hypothesis section (pages 9-10) |
| The fourth suggestion, in the methodology section, it is necessary to determine the sampling technique used, besides that to provide better value ... | Thank you for the comment. Although the sampling procedure was already included in the original manuscript, it was incorrectly placed in the Participants section - we moved it to the Research Design and Sampling Procedures section and further supplemented it, as you can see at the beginning of the section (p.11-12). |
| Researchers need to add questionnaire statements in tabular form so that they can be easily read. | Thank you for your insight. In the appendix (supplementary documents), we have included a summary of all the survey questions analysed in a tabular format. |
| The fifth suggestion, in the research results section, there should be validity and reliability testing so that the data used can be relied on, at least the indicator part of each variable. | Thank you for the feedback, we would like to inform you that Cronbach's alphas, as a reliability indicator, were included in the Instruments section, after the presentation of all questionnaires. The reliability of the EI scales obtained as a result of PCA is also reported there. In order to avoid misunderstandings, we now emphasize in the text that the reliability values were calculated on the data of the present research. We also included the reliability of the EI scales in the table showing the results of the PCA in the Supporting Information. The purpose of this research is not to validate the AES questionnaire (it was validated in Hungarian language), we used PCA as a preliminary processing step, as a data reduction procedure, and therefore we only published its results in the Supporting Information, and thus the main text does not discuss the correlations of the EI scales with the other relevant variables as a validation process. However, it can be seen that the correlations of the scales were as expected. |
| The sixth suggestion, in the discussion section, it is highly recommended to discuss each influence relationship so that the results and discussion can be interpreted properly. | Thank you for your valuable insight. We have revised the discourse to reflect your feedback, ensuring all effects and logical connections emerged from our research are clearly identified, analysed and described |
| The seventh suggestion, the conclusion should be first and then the implication, the conclusion is a summary of the discussion while the implication is the impact after this research is completed. | Thank you for your feedback. Based on your suggestion, we have adjusted the order of the sections in the text accordingly. |

**Reviewer4**

| Reviewers' Comment | Authors revision |
| --- | --- |
| Limitations: The cross-sectional design limits the ability to infer causal relationships. This could be addressed in future research by incorporating longitudinal designs to observe changes over time. | Thank you for your insights. According to your suggestions, we have recreated and extended the limitations and future implications (in conclusion) section. (First limitation reflects the cross sectional design). In the future we are keen to make a longitudinal design as we are also interested in repeated measures SEM. |
