# Supplementary material for: The mediating role of workplace milieu resources on the relationship between emotional intelligence and burnout among leaders in social care
Source: PLoS One. 2025 Jan 31;20(1):e0317280. doi: 10.1371/journal.pone.0317280 (PMC11785285; doi:10.1371/journal.pone.0317280)
Supplement: Attachment — Submitted filename: Response to Reviewers.docx [file pone.0317280.s007.docx]

**Dear Editor and Reviewers,**

We sincerely appreciate your valuable comments and insights on our manuscript. We have thoroughly reviewed all your suggestions and made comprehensive amendments to enhance the document. We believe these revisions have significantly improved its quality. The changes proposed by the editor and reviewers are detailed in the table format required in the PLOS ONE Revision letter.

**Below are our responses to the Editor comments**

| **Editor’s Comment** | **Authors revision** |
| --- | --- |
| (1) The abstract is required for data collection (study setting of Hungary's population and sampling technique). Data analysis using statistical analysis for descriptive analysis (frequency, percentage, mean, and standard deviation) and hypothesis testing using inferential statistics are needed for justification. | Thank you very much for your comment. We have revised and rewritten the abstract according to the required format, added information and suggested subsections (also aligned with the comments of Reviewers 1 & 4) |
| (2) Please add all contents of the ethical statement in the methodology: A comprehensive, multifaceted questionnaire was distributed to social care leaders at Semmelweis University in Budapest, Hungary, following ethical approval from the university's research review board (Ethical Approval of Research Semmelweis University Regional and Institutional Committee of Science and Research Ethics SE RKEB: 61/2019). Participants were asked to complete the questionnaire on paper at the conclusion of their training sessions. The questionnaire was in Hungarian, and participation was voluntary, with the option to withdraw at any time without consequences. Data was collected using anonymous pseudocodes. The recruitment period spanned from April 1, 2019, to November 30, 2019. During data collection, all adult participants enrolled in the social care management training at Semmelweis University were provided with a written consent form at the commencement of the questionnaire. Participation was voluntary and anonymous. They were informed that the processed results, containing anonymized individual data, would be published in scientific and educational publications. | Thank you for your comment we have restructured the "**Research design and sampling procedures"** & "**Ethics Statement"** sections and added all the required information. |
| (3) Kindly use the findings instead of our findings | Thank you for your comment. We have replaced “***our*** “findings with “***the”*** findings**.** |
| (4) Kindly check Figure on page 49, b2 and d, if it needs to be replaced b2 to d and d to be b2. | Thank you for the suggestion. We checked the labelling, and it was as we intended. Our reasoning is: we wanted to keep the labelling design commonly used in mediation analyses, i.e. regression lines originating from the predictor and leading to the mediator(s) are commonly labelled with “a”, and regression lines originating from the mediators and leading to the outcome variable are commonly labelled as “b”. Finally, the regression line between the two mediators is labelled as “d”. This way, a1 and b1 are the lines leading to and originating from the first mediator, and a2 and b2 are connected to the second mediator. |

**Authors Comments to the Reviewers**

**Reviewer1**

| **Reviewers' Comment** | **Authors revision** |
| --- | --- |
| Please check the abstract format of PLOS ONE and ensure it includes background, methods, results, and a conclusion. Methods in the abstract are also necessary for data collection and analysis.. | Thank you very much for your comment. We have revised and rewritten the abstract according to the required format and suggested subsections (also aligned with the comments of the Editor & Reviewer 4) |

**Reviewer2**

| **Reviewers' Comment** | **Author(s) revision** |
| --- | --- |
| The revised version is acceptable. However, kindly check for plagiarism and AI Writing similarity to be less than 15%, if any, for typographical or grammatical errors. | Thank you for your comment. We have re-verified the information with https://detecting-ai.com/, which indicated a rate of 6.8%, Grammarly 7% while justdone.ai suggested 74%. We would like to emphasize that we are not native speakers and therefore employed Grammarly, DeepL, and support from the university's lecture office for grammar and semantic checks. However, we wish to clarify that no AI was used to generate ideas or compose the text. |

**Reviewer4**

| **Reviewers' Comment** | **Authors revision** |
| --- | --- |
| recommend increasing the introduction and literature review to include fully defined variables and more detailed hypothesis development based on relevant research to strengthen the study's foundation. The introduction should state the research purpose and provide specific research questions to help readers comprehend the study's goals. | Thank you for your valuable feedback. We have revised, rewrote and reorganized the **Introduction** and **Literature Review** sections to enhance clarity, flow, and detail, ensuring that all variables are fully defined. This provides a solid foundation for the **Research Objectives and Hypothesis Development** section. In the introduction, we emphasized the research purpose and included questions to guide the reader more effectively. |
| Clarifying sampling, statistical analysis, and inferential approaches (e.g., PLS-SEM and JASP for serial mediation analysis) in the methods section would improve transparency | Thank you for the suggestions, we expanded the Methods section with details of sampling and descriptive statistics. The final version of the description of the mediations are as follows:  *„…the serial mediation models included only manifest variables calculated from the questionnaire responses (commonly called Path Analysis or the structural part of CB-SEM) and they were examined in a saturated model, which is one of the accepted approaches in the field of the current research []. In the saturated model, the point estimates of the regression coefficients and the indirect effects are the same, regardless of which regression calculation method or structural equation modelling is used (in this study, for reasons of convenience, Maximum Likelihood modelling was used). Standard Errors and Confidence intervals were obtained by percentile bootstrap for regression coefficients and indirect effects as well. Lavaan syntax of the saturated mediation models can be found in S3.”*  We hope that this description adequately expresses that we did not use PLS-SEM, but a CB-SEM, in which we tested a saturated model and used the variables in manifest form, with their scale scores calculated in advance (i.e. we used a structural model). The reliability of the questionnaires was described during the presentation of the questionnaires but we also added the Cronbach’s alpha values to Table 1. |
| Adding a methodologies and analytic approaches overview to the abstract will help set reader expectations. | Thank you very much for your comment. We have revised and rewritten the abstract according to the required format and suggested subsections (also aligned with the comments of the Editor & Reviewer 1) |
| Validity and reliability testing for measurement tools and better significance indications in tables would strengthen results. | Thank you for the suggestion. Reliability measures (Cronbach’s alphas) are reported in the Methods section after the description of each questionnaire. However, we also included these values to the correlation table. We hope that this solution will meet the desired reporting standards.  Significance is indicated with p-values, with asterisks, bolding, and confidence intervals. Now we also mentioned in the notes the meaning of bold. We struggle to find any better significance indications. |
| The explanation should structure the analysis around the hypotheses, detail each relationship, and address strategic planner implications to improve interpretability and application. | -Thank you for your insightful comment. We have provided specific references to the relevant lines for each hypothesis in the hypotheses section. We have mirrored this approach in the discussion section, ensuring that all relationships are thoroughly articulated.  -Thank you very much for your comment. According to the comments, we have rewritten, restructured, and extended the section, which is newly named “**Theoretical Implications and Limitations.”** |
| Though Reviewer 2 appreciates the clarity on translation and proofreading processes, minimizing automated tool use may minimize AI-similarity concerns. | Thank you for your comment. We have re-verified the information with https://detecting-ai.com/, which indicated a rate of 6.8%, Grammarly suggested 7% while justdone.ai suggested 74%. We would like to emphasize that we are not native speakers and therefore employed Grammarly, DeepL, and support from the university's lecture office for grammar and semantic checks. However, we wish to clarify that no AI was used to generate ideas or compose the text. |
| Reviewer 4's recommendation to acknowledge cross-sectional design limitations and propose longitudinal research could improve the study's causal inferences. | Thank you very much for your comment we extended the **Theoretical implications and Limitations** as follows: “*Firstly, this study was based on a cross-sectional design. As a result, it cannot reflect the evolving dynamics and interactions between the studied phenomena, and reverse or reciprocal causal relationships cannot be analysed. To address these limitations, future research could employ a longitudinal design to examine changes and interactions among emotional intelligence, workplace resources, and burnout over time.”* |
