# Supplementary material for: The mediating role of workplace milieu resources on the relationship between emotional intelligence and burnout among leaders in social care
Source: PLoS One. 2025 Jan 31;20(1):e0317280. doi: 10.1371/journal.pone.0317280 (PMC11785285; doi:10.1371/journal.pone.0317280)
Supplement: Attachment — Submitted filename: Response to Reviewers.docx [file pone.0317280.s008.docx]

**Dear Editor and Reviewer,**

We sincerely appreciate your valuable comments and insights on our manuscript. We have thoroughly reviewed your suggestion and made comprehensive amendments to enhance the document. We believe the revision has significantly improved its quality. The change proposed by the editor and reviewer are detailed in the table format required in the PLOS ONE Revision letter.

**Below are our responses to the Editor and Reviewer comments**

| **Editor Comment** | **Authors revision** |
| --- | --- |
| .  **Acceptable; however, the recruitment period spanned from April 11th to November 30th, 2019. The results are reflected in 2019 (not 2024 as of the current year). If COVID-19 may affect the results of the study "The Mediating Role of Workplace Milieu Resources on the Relationship between Emotional Intelligence and Burnout among Leaders in Social Care," Please discuss whether further studies are recommended in this inquiry** | ***Thank you for this valuable observation. Indeed, the interventions to tackle the COVID-19 pandemic, may have exacerbated burnout and impacted emotional intelligence among social care leaders. However, research on this subject is sparse in the local setting. The authors decided to reflect on this observation by revising the "Theoretical Implications and Limitations section" to add a final limitation paragraph of the manuscript. We hope that the added text meets the Editor's and the Reviewer's expectations.***  ***The added limitation paragraph is as follows:***  *" The recruitment period for this study (April 11th to November 30th, 2019) predates the global COVID-19 pandemic, which has drastically reshaped workplace dynamics, particularly in the social and healthcare sectors. Although Hungary largely resumed in-person work—aligned with the personal nature of social work—the pandemic introduced profound challenges that could exacerbate burnout and workplace demands. For example, Dima, Schmitz, and Simon's (2021) research found elevated stress levels among Romanian social workers during the pandemic associated with increased burnout (72). Whereas McCoyd and Colleagues suggested that concerning the COVID-19 pandemic, social workers demonstrated resilience, adapting to increased demands, integrating new technologies, and addressing service gaps despite experiencing symptoms of burnout (73). These findings suggest that the structural and psychological impacts of the pandemic may have influenced the interplay between emotional intelligence, workplace resources, and burnout prevention among leaders in social care. A follow-up study incorporating post-pandemic data will address these limitations, focusing on workplace demands, structural changes, and the evolving roles of emotional intelligence and resources in mitigating burnout. This future research aims to understand better the social care sector's changing dynamics and practical strategies for leader support in a post-pandemic context.”* |
|  |  |

| **Reviewer 1 Comment** | **Authors revision** |
| --- | --- |
| .  **Acceptable; however, the recruitment period spanned from April 11th to November 30th, 2019. The results are reflected in 2019 (not 2024 as of the current year). If COVID-19 may affect the results of the study "The Mediating Role of Workplace Milieu Resources on the Relationship between Emotional Intelligence and Burnout among Leaders in Social Care," Please discuss whether further studies are recommended in this inquiry.** | ***Thank you for this valuable observation. Indeed, the interventions to tackle the COVID-19 pandemic, may have exacerbated burnout and impacted emotional intelligence among social care leaders. However, research on this subject is sparse in the local setting. The authors decided to reflect on this observation by revising the "Theoretical Implications and Limitations section" to add a final limitation paragraph of the manuscript. We hope that the added text meets the Editor's and the Reviewer's expectations.***  ***The added limitation paragraph is as follows:***  *" The recruitment period for this study (April 11th to November 30th, 2019) predates the global COVID-19 pandemic, which has drastically reshaped workplace dynamics, particularly in the social and healthcare sectors. Although Hungary largely resumed in-person work—aligned with the personal nature of social work—the pandemic introduced profound challenges that could exacerbate burnout and workplace demands. For example, Dima, Schmitz, and Simon's (2021) research found elevated stress levels among Romanian social workers during the pandemic associated with increased burnout (72). Whereas McCoyd and Colleagues suggested that concerning the COVID-19 pandemic, social workers demonstrated resilience, adapting to increased demands, integrating new technologies, and addressing service gaps despite experiencing symptoms of burnout (73). These findings suggest that the structural and psychological impacts of the pandemic may have influenced the interplay between emotional intelligence, workplace resources, and burnout prevention among leaders in social care. A follow-up study incorporating post-pandemic data will address these limitations, focusing on workplace demands, structural changes, and the evolving roles of emotional intelligence and resources in mitigating burnout. This future research aims to understand better the social care sector's changing dynamics and practical strategies for leader support in a post-pandemic context.”* |
|  |  |
